# Prevalence of Diabetes in the Republic of Ireland: Results from the National Health Survey (SLAN) 2007

**DOI:** 10.1371/journal.pone.0078406

**Published:** 2013-10-16

**Authors:** Kevin P. Balanda, Claire M. Buckley, Steve J. Barron, Lorraine E. Fahy, Jamie M. Madden, Janas M. Harrington, Ivan J. Perry, Patricia M. Kearney

**Affiliations:** 1 Institute of Public Health in Ireland, Dublin, Ireland; 2 Department of General Practice, University College Cork, Cork, Ireland; 3 Department of Epidemiology & Public Health, University College Cork, Cork, Ireland; Sapienza University of Rome, Italy

## Abstract

**Background:**

Current estimates of diabetes prevalence in the Republic of Ireland (RoI) are based on UK epidemiological studies. This study uses Irish data to describe the prevalence of doctor-diagnosed diabetes amongst all adults aged 18+ years and undiagnosed diabetes amongst those aged 45+ years.

**Methods:**

The survey of lifestyle attitudes and nutrition (SLAN) 2007 is based on a nationally representative sample of Irish adults aged 18+ years (n = 10,364). Self-reported doctor-diagnosed diabetes was recorded for respondents in the full sample. Diabetes medication use, measured height and weight, and non-fasting blood samples were variously recorded in sub-samples of younger (n = 967) and older (n = 1,207) respondents.

**Results:**

The prevalence of doctor-diagnosed diabetes amongst adults aged 18+ years was 3.5% (95% CI 3.1% - 3.9%). After adjustment for other explanatory variables; the risk of self-reported doctor-diagnosed diabetes was significantly related to age (p < 0.0001), employment status (p = 0.0003) and obesity (p = 0.0003). Amongst adults aged 45+ years, the prevalence of doctor-diagnosed diabetes was 8.9% (95% CI 7.3% -10.5%) and undiagnosed diabetes was 2.8% (95% CI 1.4% - 4.1%). This represented 31.2% of diabetes cases in this age group.

**Conclusion:**

Notwithstanding methodological differences, these prevalence estimates are consistent with those in the UK and France. However, the percentage of undiagnosed cases amongst adults aged 45+ years appears to be higher in the RoI. Increased efforts to improve early detection and population level interventions to address adverse diet and lifestyle factors are urgently needed.

## Introduction

Diabetes is responsible for significant premature deaths, reduced quality of life and costs to the health system and the economy [1]. The prevalence of diabetes is increasing worldwide [2] and changes in lifestyle and obesity levels contribute to the rise in diabetes prevalence, especially in affluent countries [3]. 

Accurate estimates of the number of people with diagnosed and undiagnosed diabetes are required for effective healthcare service planning. In the Republic of Ireland (RoI), prevalence estimates are available for particular age groups in primary care settings [4,5]. While many developed countries provide trends in national prevalence rates [6,7], current national figures for the RoI are based on Oral Glucose Tolerance Test (OGTT) results from population-based epidemiological studies in the UK [8]. 

In the past, an OGTT was the gold standard for diagnosis of diabetes [9]. Since 2009, however, the American Diabetes Association (ADA) endorses the use of HbA1c for the diagnosis of diabetes [10]. 

Based on an Irish national health survey including HbA1c measurements, the aim of the study was to develop a logistic regression model and use it to describe variation in the prevalence across the RoI of doctor-diagnosed diabetes amongst all adults and the prevalence of undiagnosed diabetes amongst adults aged 45+ years.

## Methods

### Ethics statement

Study protocols were given ethical approval by the Research Ethics Committee of the Royal College of Surgeons in Ireland.

### SLAN 2007 survey

SLAN 2007 was a cross-sectional survey of health and lifestyle in the RoI [11]. From 16,681 adults selected from the country’s GeoDirectory [12], 10,364 completed questionnaires were obtained from a nationally representative sample of adults aged 18+ years living in private households in the RoI (response rate 62.1%).

The survey comprised face-to-face detailed health and lifestyle interviews administered by trained social interviewers as well as two measurement sub-samples: anthropometric measures in a sub-sample of 967 respondents aged 18-44 years and a more detailed physical examination of a sub-sample of 1,207 respondents aged 45+ years. 

The full sample distribution and the sub-sample distributions were calibrated to population totals using the Quarterly National Household Survey (QNHS) [13] and Census 2006 [14]. 

### Measurements

All survey respondents were asked if they had diabetes in the last 12 months (yes/no) and, if so, whether it had been diagnosed by a doctor. Height, weight and waist circumference were measured in the “younger sub-sample” (respondents aged 18-44 years). Respondents in the “older sub-sample” (those aged 45+ years) had their weight and height measured, were asked if they were currently taking medication for diabetes (yes/no), and provided non-fasting blood samples. Blood for glycated haemoglobin (HbA1c) was collected in a whole blood ethylenediaminetetraacetic acid (EDTA) tube and measured using an immunoturbidimetric method. 

The following socio-demographic and lifestyle variables were available for all respondents: sex, age (18-34 years, 35-44 years, 45-54 years, 55-64 years, 65-74 years, 75+ years), ethnicity (White, Non-white), calculated BMI (Underweight/Normal [BMI < 25 kg/m^2^], Overweight [BMI 25-29.9 kg/m^2^], Obese [BMI 30+kg/m^2^)], physical activity (Low, Moderate, High) based on the IPAQ [15], cigarette smoking (Never smoked, Former smoker, Current smoker), alcohol consumption (Never/Monthly or less/2-4 times per month, 2-3 times per week, 4+ times per week), fruit and vegetable consumption (

< 5 per day, 5+ per day), highest level of education (Primary level, Secondary level, Third level), employment status (Employed, Unemployed, Economically inactive), social class (SC 1-2 [Professional and managerial], SC 3-4 [Non-manual and skilled manual], SC 5-6 [Semi-skilled and unskilled], Unclassified) and area deprivation. Standard age categories, adapted where necessary to take onto account sample sizes, and standard BMI categories were selected to facilitate international comparisons. 

### Statistical analyses

Estimates of the prevalence of undiagnosed diabetes amongst those aged 45+ years were based on the sub-sample of older respondents with measurements of bloods. In this sub-sample, a respondent was defined as having undiagnosed diabetes if they did not self-report a doctor diagnosis, they did not currently use diabetes medication, but had HbA1c level >= 6.5%, the ADA cut-point [10]. Population prevalence of diabetes (diagnosed and undiagnosed) amongst adults aged 45+ years was taken to be the sum of the doctor-diagnosed rate and the undiagnosed rate.

Measured BMI was only available for 2,170 respondents from the two sub-samples; the remaining BMIs were calculated using self-reported height and weight. Because of small sample sizes in this study it was not possible to fully adjust self-reported BMI. Instead, all self-reported BMIs within a (sex, age) category were multiplied by an adjustment factor defined as the mean measured BMI in that (sex, age) category divided by the mean self-reported BMI in that (sex, age) category. This ensured that, for each (sex, age) category, the mean adjusted self-reported BMI matched the mean measured BMI in that category. The adjustment factors were larger for females and larger for older respondents, ranging from 1.03 to 1.08. 

A stepwise selection logistic regression procedure (entry p-value = 0.05, exit p-value = 0.05) was used to develop an explanatory model of doctor-diagnosed diabetes amongst adults aged 18+ years [16]. All variables listed in the measurement section above were included; no variables were removed during the procedure. PROC SURVEYLOGISTIC (SAS Version 9.2), which takes into account clustering in the sample design, was used to develop the model. 

An approximate confidence interval, amongst adults aged 45+ years, for the male:female ratio of the proportion of cases that are undiagnosed was based on data about undiagnosed cases from the sub-sample of older respondents (n = 1,207) and data on diagnosed cases from the larger full sample of 45+ year olds (n = 5,147). A first order Taylor series expansion was used to incorporate the standard errors for the male:female ratios of undiagnosed cases and male:female ratio of diagnosed cases into the approximate confidence interval.

## Results

The response rate for the full survey of adults aged 18+ years was 62% (n=10,364). The response rate for the younger sub-sample (respondents aged 18-44 years) was 58% (n=967) and the response rate for the older sub-sample (respondents aged 45+ years) was 66% (n=1,207). Of the 1,207 respondents in the older sub-sample, 1,132 adequately completed the main survey questionnaire and provided a blood sample for HbA1c analysis. 

The socio-demographic profile of the sample of 10,364 responses comprised a nationally representative sample of adults aged 18+ years living in private households in the RoI; the sex-age profile of the sample is given in Table 1.

**Table 1:** Weighted sex-age sample sizes of the SLAN 2007 survey (Republic of Ireland, 2007).

Age	Sex		Total
	Males	Female	
18-34 years	1,965.1	1,907.2	3,862.3
35-44 years	996.1	979.0	1,975.0
45-54 years	836.6	837.5	1,674.0
55-64 years	669.0	670.2	1,339.2
65-74 years	423.9	461.1	885.0
75+ years	250.5	337.9	628.5
Total	5,131.1	5,232.9	10,364.0

A total of 0.9% of respondents in the older sub-sample (aged 45+ years) said they did not have doctor-diagnosed diabetes but were currently taking diabetes medication. These respondents were reclassified as having doctor-diagnosed diabetes. The sex-age specific percentages of respondents aged 45+ years in the full sample with self-reported doctor-diagnosed diabetes were adjusted accordingly. Because diabetes medication was not recorded in the younger sub-sample (aged 18-44 years), no such adjustment was made there. 

### Prevalence of doctor-diagnosed diabetes amongst adults aged 18+ years

**Figure 1:**
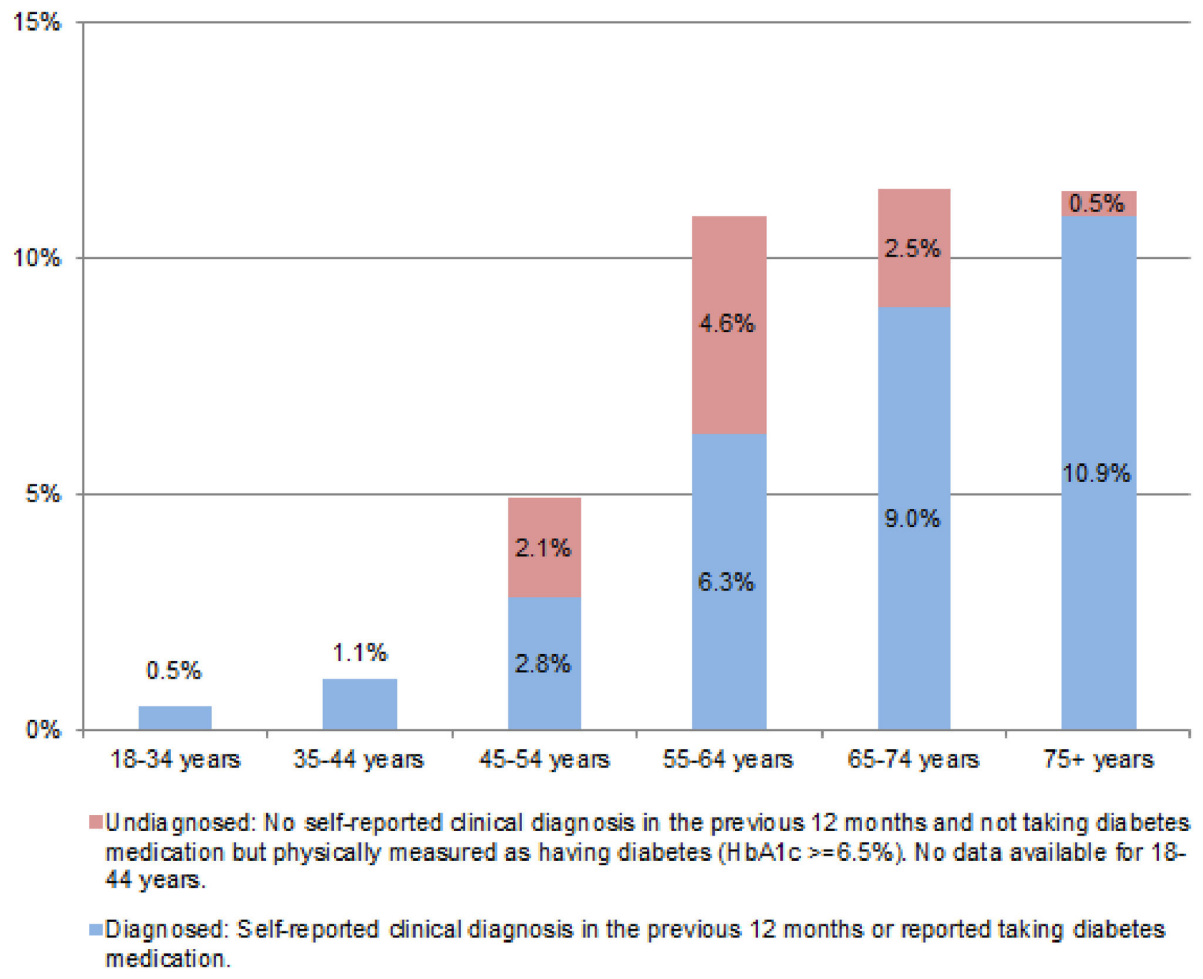
Prevalence of diabetes by age, Republic of Ireland, 2007.

It is estimated that 3.5% (95% CI = 3.1% - 3.9%) of adults aged 18+ years had doctor-diagnosed diabetes in 2007. The prevalence of doctor-diagnosed diabetes amongst males and females were similar amongst those aged 18-44 years (p > 0.1) and those aged 45+ years (p > 0.2). Doctor-diagnosed diabetes was significantly more common among older respondents; rising from 0.7% (95% CI 0.5% - 0.9%) amongst adults aged 18-44 years to 6.1% (95% CI 5.3% - 6.9%) amongst adults aged 45+ years (see Figure 1).

The explanatory model derived from the stepwise regression procedure explained significant deviance (Likelihood Ratio Chi-squared test = 282.4810, df = 9, p <.0001) and showed no evidence of lack of fit (Hosmer and Lemeshow Chi Square test = 4.2235, df=8, p=0.8364). Age, employment status and BMI entered the model in that order before the procedure stopped. After adjustment for all other explanatory variables; the risk of self-reported doctor-diagnosed diabetes was significantly related to age (Chi-squared = 79.7686, df = 5, p < 0.0001), employment status (Chi-squared = 16.2037, df = 2, p = 0.0003) and obesity (Chi-squared = 16.0525, df = 2, p = 0.0003). Table 2 identifies subgroups of respondents who were at significantly increased independent risk of self-reported doctor-diagnosed diabetes. After adjustment for all other explanatory variables; all age groups were more likely than those aged 18-34 years to have doctor-diagnosed diabetes; economically inactive respondents were twice as likely as those who were employed to have doctor-diagnosed diabetes (OR = 2.02, 95% CI = 1.39 - 2.92), and obese respondents were twice as likely as those with underweight/normal weight (OR = 2.15, 95% CI = 1.40 - 3.29) to have doctor-diagnosed diabetes.

**Table 2:** Odds ratios for the explanatory model of self-reported doctor-diagnosed diabetes^1^ amongst adults aged 18+ years (Republic of Ireland, 2007)

		Odds ratio (OR)	95% CI for OR	Pr > ChiSq
Age	18-34 years (reference)	1.00		
	35-44 years	2.22	1.03 - 4.80	0.04
	45-54 years	4.65	2.25 - 9.60	<0.0001
	55-64 years	9.90	4.86 - 20.14	<0.0001
	65-74 years	10.12	5.09 - 20.11	<0.0001
	75+ years	11.41	5.57 - 23.37	<0.0001
Employment	Employed (reference)	1.00		
	Economically inactive	2.02	1.39 - 2.92	<0.001
	Unemployed	0.40	0.09 - 1.75	0.23
BMI	Underweight/Normal (reference)	1.00		
	Overweight 25-29.99	1.35	0.88 - 2.08	0.18
	Obese 30+	2.15	1.40- 3.29	<0.001

^1^ Does not include respondents who did not report a doctor-diagnosed case but reported, in the older sub-sample, that they were currently taking diabetes medications.

### Prevalence of diagnosed and undiagnosed diabetes amongst adults aged 45+ years

Table 3 describes the prevalence of doctor-diagnosed and undiagnosed diabetes amongst adults aged 45+ years. Overall, 31.2% of all diabetes cases amongst adults aged 45+ years were undiagnosed. The proportion of diabetes cases that were undiagnosed was particularly high amongst middle aged adults (aged 45-64 years). While the proportion of diabetes cases that were undiagnosed was higher amongst males (36.7%) than amongst females (23.5%), this difference was not statistically significant (approx. 95% CI for ratio 0.89 - 4.31). 

**Table 3:** Prevalence of doctor-diagnosed and undiagnosed diabetes amongst adults age 45+ years, by sex and age (Republic of Ireland, 2007)^1^

	Total (diagnosed & undiagnosed)^2^ (95% CI)	Doctor-diagnosed^2^ (95% CI)	Undiagnosed^3^ (95% CI)	Percentage of cases undiagnosed
Sex				
Males	10.8% (8.2% -13.4%)	6.8% (5.7% -7.9%)	4.0% (1.6% -6.3%)	36.7%
Females	7.1% (5.3% -8.9%)	5.4% (4.3% -6.6%)	1.7% (0.3% -3.0%)	23.5%
Age (Persons)				
45+ years	8.9% (7.3% -10.5%)	6.1% (5.3% -6.9%)	2.8% (1.4% -4.1%)	31.2%
45-54 years	4.9% (3.3% -6.6%)	2.8% (1.9% -3.7%)	2.1% (0.8% -3.5%)	43.3%
55-64 years	10.9% (6.9% -14.9%)	6.3% (4.6% -8.0%)	4.6% (1.0% -8.2%)	42.3%
65–74 years	11.5% (8.0% -15.0%)	8.9% (6.9% -11.0%)	2.5% (0.0% -5.3%)	21.9%
75+ years	11.4% (8.5% -14.3%)	10.9% (8.2% -13.6%)	0.5% (0.0% -1.6%)	4.6%
Age (Males)				
45+ years	10.8% (8.2% -13.4%)	6.8% (5.7% -7.9%)	4.0% (1.6% -6.3%)	36.7%
45-54 years	5.4% (2.8% -7.9%)	2.5% (1.2% -3.8%)	2.9% (0.7% -5.1%)	53.6%
55-64 years	16.6% (9.2% -24.0%)	8.7% (6.2% -11.3%)	7.8% (0.9% -14.8%)	47.3%
65–74 years	12.4% (9.2% -15.5%)	10.7% (8.3% -13.1%)	1.7% (0.0% -3.8%)	13.8%
75+ years	10.5% (6.3% -14.6%)	9.2% (5.9% -12.5%)	1.3% (0.0% -3.8%)	12.3%
Age (Females)				
45+ years	7.1% (5.3% -8.9%)	5.4% (4.3% -6.6%)	1.7% (0.3% -3.0%)	23.5%
45-54 years	4.5% (2.6% -6.4%)	3.1% (1.9% -4.3%)	1.4% (0.0% -2.9%)	31.3%
55-64 years	5.3% (2.8% -7.9%)	3.9% (1.9% -5.8%)	1.5% (0.0% -3.1%)	27.6%
65–74 years	10.6% (4.8% -16.5%)	7.4% (4.3% -10.6%)	3.2% (0.0% -8.1%)	30.3%
75+ years	12.1% (8.1% -16.1%)	12.1% (8.1% -16.1%)	0.0% (0.0% -0.0%)	0.0%

^1^ Some column totals will differ from sum of corresponding entries because of rounding error.

^2^ Based on measures collected in the full study and older sub-sample.

^3^ Based on measures collected in older sub-sample only.

## Discussion

This is the first study to use a representative sample of the Irish population to estimate the prevalence of doctor-diagnosed diabetes amongst adults aged 18+ years and undiagnosed diabetes amongst adults aged 45+ years.

The SLAN 2007 survey is the most recent national health survey available that covers all adult ages. Nevertheless, the survey is six years old and RoI’s risk factor profile is likely to have deteriorated. Comparison of SLAN 2007 and Census 2011 suggest that, between 2007 and 2011, the RoI’s population profile has aged and relatively more adults aged 18+ years are either unemployed or economically inactive. It is likely that prevalence estimates for 2007 underestimate the prevalence in 2013. 

While the use of HbA1c to diagnose diabetes has been endorsed by an International Expert Committee and the ADA, some controversy exists concerning the suitability of using HbA1c for diagnosis [17]. However, HbA1c is considered acceptable for the purposes of reporting prevalence estimates and has been used previously in this context [6]. Ideally, we would report diabetes prevalence estimates based on both HbA1c and OGTT results, if available. 

 A further limitation of the study is the lack of a range of physical measurements on the full sample; influencing the accuracy of prevalence estimates and BMI and necessitating a number of adjustments.

Diabetes prevalence studies use different definitions and methodology; limiting the direct comparisons that can be made. While the use of HbA1c levels to identify diabetes cases is aligned with the ADA guidelines, the absence of OGTT in the survey means it is difficult to make comparisons with earlier studies using OGTT and international studies using OGTT. Using a combination of self-reported diagnoses and HbA1c results, Bonaldi et al. reported a prevalence rate of 12% (95% CI = 9.1%-15.7%) in a 55-74 year cohort in France in 2007 [6]. Using a combination of self-reported diagnoses and fasting plasma glucose measurement, Pierce et al. reported a prevalence of diabetes of 9.1% (95% CI = 8.3% - 9.9%) in the 52-79 year age group of the English Longitudinal Study of Ageing (ELSA) 2004/2005 [7]. While acknowledging difference in methodology, our prevalence estimates amongst older adults are consistent with estimates in UK and France. 

The higher proportion of diabetes cases that were undiagnosed amongst middle aged adults (aged 45-64 years) amongst males (although not statistically significant) might be explained by corresponding variation in the use of general practice services. 

While it is generally agreed that widespread screening for diabetes is inappropriate, targeted screening of high-risk patients has been shown to be cost-effective [18,19]. Although McHugh et al. reported that nearly all (93%) general practitioners in the RoI report screening high-risk individuals for diabetes [20], almost one third (31.2%) of diabetes cases amongst adults aged 45+ years in the our study were undiagnosed. While this figure is considerably higher than percentages of cases that were undiagnosed reported in the UK (18.5%) and France (25%) as discussed above, it is consistent with other studies. For example, using HbA1c criteria, Hayes et al. found that 28% of 60-74 year old patients with diabetes from the practice population in an affluent suburb of Newcastle-Upon-Tyne were undiagnosed [21]. The Association of Public Health Observatories (APHO) prevalence model estimated that across England, approximately 27% of adults with diabetes are currently undiagnosed [22]. The DECODE study group found that the percentage of persons with diabetes that were undiagnosed varied by age and sex; ranging from 50% amongst men aged 60-69 years to 42% amongst women aged 60-69 years [23].

A large systematic analysis of national health examination surveys and epidemiological studies recently concluded that rising global diabetes is driven by population growth, ageing and increasing age-specific prevalence rates [24]. The findings of this study highlight the urgent need to strengthen efforts to prevent, detect and manage diabetes. National policies and strategies emphasise prevention and include targets for improvement in population levels of risk factors through prevention programmes that address social, environmental and other issues that influence the development of diabetes [25-27]. Targeted case finding is needed to identify undiagnosed diabetes and reduce future health damage. General practitioners need to be vigilant with screening for dysglycaemia, particularly older males, and obese or hypertensive individuals.
