# Correction: Prevalence of Diabetes in the Republic of Ireland: Results from the National Health Survey (SLAN) 2007

**DOI:** 10.1371/journal.pone.0099471

**Published:** 2014-06-03

**Authors:** The PLOS ONE Staff

There is an error in the second sentence of the “Results” sub-heading under the "Abstract" section. The correct sentence for this section is:

"Amongst adults aged 45+ years, the prevalence of doctor-diagnosed diabetes was 6.1% (95% CI 5.3% - 6.9%) and undiagnosed diabetes was 2.8% (95% CI 1.4% - 4.1%)."
